# A Household-Based Study of Contact Networks Relevant for the Spread of Infectious Diseases in the Highlands of Peru

**DOI:** 10.1371/journal.pone.0118457

**Published:** 2015-03-03

**Authors:** Carlos G. Grijalva, Nele Goeyvaerts, Hector Verastegui, Kathryn M. Edwards, Ana I. Gil, Claudio F. Lanata, Niel Hens, for the RESPIRA PERU project

**Affiliations:** 1 Department of Health Policy, Vanderbilt University School of Medicine, Nashville, TN, United States of America; 2 Department of Pediatrics, Vanderbilt University School of Medicine, Nashville, TN, United States of America; 3 Interuniversity Institute for Biostatistics and Statistical Bioinformatics, Hasselt University, Diepenbeek, Belgium; 4 Centre for Health Economics Research and Modeling Infectious Diseases, and Centre for the Evaluation of Vaccination, Vaccine & Infectious Disease Institute, University of Antwerp, Wilrijk, Belgium; 5 Instituto de Investigación Nutricional, Lima, Peru; Georgia State University, UNITED STATES

## Abstract

**Background:**

Few studies have quantified social mixing in remote rural areas of developing countries, where the burden of infectious diseases is usually the highest. Understanding social mixing patterns in those settings is crucial to inform the implementation of strategies for disease prevention and control. We characterized contact and social mixing patterns in rural communities of the Peruvian highlands.

**Methods and Findings:**

This cross-sectional study was nested in a large prospective household-based study of respiratory infections conducted in the province of San Marcos, Cajamarca-Peru. Members of study households were interviewed using a structured questionnaire of social contacts (conversation or physical interaction) experienced during the last 24 hours. We identified 9015 reported contacts from 588 study household members. The median age of respondents was 17 years (interquartile range [IQR] 4–34 years). The median number of reported contacts was 12 (IQR 8–20) whereas the median number of physical (i.e. skin-to-skin) contacts was 8.5 (IQR 5–14). Study participants had contacts mostly with people of similar age, and with their offspring or parents. The number of reported contacts was mainly determined by the participants’ age, household size and occupation. School-aged children had more contacts than other age groups. Within-household reciprocity of contacts reporting declined with household size (range 70%-100%). Ninety percent of household contact networks were complete, and furthermore, household members' contacts with non-household members showed significant overlap (range 33%-86%), indicating a high degree of contact clustering. A two-level mixing epidemic model was simulated to compare within-household mixing based on observed contact networks and within-household random mixing. No differences in the size or duration of the simulated epidemics were revealed.

**Conclusion:**

This study of rural low-density communities in the highlands of Peru suggests contact patterns are highly assortative. Study findings support the use of within-household homogenous mixing assumptions for epidemic modeling in this setting.

## Introduction

Understanding the transmission of infectious diseases in specific populations is crucial for the tailored design of effective strategies for disease prevention and control. Transmission patterns of infections among humans are closely related to patterns of social interaction.[1–3] Yet until recently, this valuable research area has received very little attention.

Studies on social mixing and contact patterns provide valuable information for mathematical models of disease transmission in specific populations and settings. Measurements of social interactions provide data to inform model parameters that were traditionally based on untested assumptions. Modeling approaches can be improved with the use of auxiliary contact data, which allows a detailed characterization of the interactions among different individuals within a given population or setting.[4–7] Although households are important components in the disease transmission process, relatively little work has been done to estimate contact networks within households.[8] Most transmission models assumed homogeneous mixing within households.[9,10] For example, Fumanelli et al used routine socio-demographic data to compute contact matrices for 26 European countries, but postulated random mixing within households to compute within-household contact matrices.[11]

Social mixing patterns may be influenced by a number of factors including socioeconomic characteristics, the physical environment and geographical location, living and working conditions, social and cultural patterns and individual lifestyle choices. These factors are expected to vary by place and time.[3] Most studies on social mixing have been conducted in urban populations of developed countries or highly populated areas.[1–3,12] However, few studies have quantified social mixing in remote rural areas of developing countries,[12–14] where the high prevalence of risk factors for disease and other factors that also facilitate disease transmission likely contribute to a high burden and severity of infectious diseases.[13,14] We sought to study social contact patterns in rural, high-altitude, low population-density communities of the Peruvian Andes.

## Methods

### Study area and population

The study was conducted in the province of San Marcos, Department of Cajamarca, in the northern highlands of Peru. Nearly all San Marcos inhabitants descend from the same ethnic group of Spaniards mixed with the local indigenous Andean population. The altitude in this province ranges from approximately 1,500 to 4,000 meters above sea level. San Marcos has a mountainous terrain and the average temperature ranges between 8 and 30°C, and varies across the different altitude settings.[13]

San Marcos’ population is comprised of separated accessible rural communities. The population is mainly low income, low educational level, with limited access to healthcare services. The estimated median age in San Marcos for 2011 was 22 years, and according to the most recent census, the average number of people per household was four.[15] The United Nations Development Program (UNDP) [16] estimated that in 2011, the life expectancy at birth was 69.96 years in San Marcos (73.99 years for Peru), the proportion of people 12–16 years old attending secondary education was 70.0% (79.9% for Peru), the proportion with secondary education among 18 years old was 26.2% (66.3% for Peru), the number of years of education among 25 years old or older was 4.9 years (8.8 years for Peru), and the monthly income was approximately US $92 (US $235 for Peru). The estimated Human Development Index (HDI) for San Marcos was 0.2523 (0.4906 for Peru). In addition, the UNDP reported that the proportion of the San Marcos population with a personal identification document was 97.1% (98.2% for Peru), the number of physicians per 10,000 inhabitants was 4.4 (18.6 for Peru), the proportion of houses with potable water and sewage was 68.3% (67.4% for Peru) and the proportion of houses with electricity was 45.1% (82.2% for Peru).[16]

Information on social contact patterns was collected in the context of the Study of Respiratory Infections in Andean Peruvian children (RESPIRA PERU). In brief, San Marcos’ households with children younger than 3 years of age (index children) were enrolled and followed through weekly household visits from May 2009 through September 2011. The median number of people living in study households was 5, and the materials of which the houses were predominantly made were typical of rural Andean settings, including dirt floors, tile roofs, and mud brick walls. Most houses used open fires or traditional stoves and wood for cooking. Most of the children (91%) in the study received health care through the public health insurance system of Peru. Most of the households’ heads worked in agriculture. Trained field workers collected information on respiratory symptoms and gathered respiratory samples when children were ill. That study enrolled children from 58 rural communities in a dynamic cohort, aiming to maintain a sample of approximately 500 children under follow-up at any given time.[13,17–19]

### Data collection

For this study, we aimed to enroll at least two households from each study community. Household selection was based on convenience and accessibility to the study field workers. A total of 114 households from 51 communities were invited and all agreed to participate in the study.

During scheduled visits, members of study households were interviewed about all contacts experienced, i.e. their encounters with different persons, from 5 am on the previous day to 5 am on the present day. Contact information for children younger than 10 years was provided by the parents. For children attending school, field workers obtained additional information from the classroom teacher. During the interviews, field workers used a structured questionnaire previously developed for assessments of social contacts in Europe and adapted after field-testing.[1] If study household members were not present at the time of the visit, up to two additional attempts to interview the members on different occasions were made. For the study assessments, a contact was defined as a conversation with another person that is physically present and no farther than 3 meters, or a physical contact involving skin-to-skin touching, e.g. a kiss or handshake (either with or without conversation).[1] Participants reported their age (self-report) and also provided information on the sex and estimated age of the contact person(s). Each contact was further characterized by location, duration and estimated frequency of contacts with the same person.[1,12] Collection of contact assessments was completed between August-October 2011. Study questionnaires are available as Supporting information.

### Ethics Statement

Among selected households, parents or guardians who had previously provided signed informed consent for young children <3 years old to participate in the RESPIRA-Peru project were informed of this related contact assessment and asked if they would agree to continue with the study, to which they gave their verbal consent. For this assessment of contact patterns, other household members from the selected households were enrolled in the study after the study procedures were explained, questions addressed and verbal informed consent was obtained. The study protocol was approved by the Vanderbilt Institutional Review Board (Nashville TN, USA), and by the Ethics Committee of the Instituto de Investigacion Nutricional, IIN (Lima, Peru).

### Data analysis

#### Number of contacts and participant characteristics

The number of contacts was regressed over the following participant characteristics: age category, sex, relationship, occupation, type of day (weekday or weekend) and household size. We used a random intercepts model (conditional model) to account for the correlation among the number of contacts of individuals belonging to the same household. Fitting the model was done using the INLA package in R. We also fitted a marginal model where the clustering within households was treated as a nuisance parameter. We used a count model in both cases. Overdispersion was allowed using a negative binomial distribution in the random intercepts model, and an overdispersed Poisson distribution was used in the marginal model. We contrasted the multivariate conditional model with univariate conditional models for all covariates. Our primary analysis focused on the number of total contacts; a secondary analysis focused on the number of physical contacts. For all statistical tests, we considered a 5% significance level.

#### Contacts by location, duration and frequency

We calculated the proportion of contacts by location, duration, frequency, type of contact and by participant's age-category.

#### Who Acquires Infection From Whom (i.e. who has contact with whom)?

To assess the patterns of contacts based on age categories, we estimated a “*who has contact with whom*” matrix using the contact data. Under the social contact hypothesis, this contact matrix can be used to inform a “*Who Acquires Infection From Whom*” (*WAIFW*) matrix in this specific study population.[4] Let *y_ijt_*, (*t* = 1,…, n*_i_*, *i* = 1,…*I*, *j* = 1,…*I*) denote the number of contacts made by participant *t* in age-category *i* with people in age-category *j*. We can then calculate the mean number of contacts made by participants in age-category *i* with people in age-category *j* as $m_{ij}=n_{i}^{-1}\sum_{t=1}^{n_{i}}y_{ijt}$. This approach was further stratified by the type of contact (e.g. physical and non-physical).

#### Exploration of household contact networks

For this assessment, households that were not completely sampled on the same day or for which at least one member did not report contacts were excluded (approximately 52.6% of study households). A contact between household members was considered reciprocal when it was reported by both household members. Household reciprocity was calculated as the fraction of reciprocal within-household contacts reporting for all individuals in households of a given size. For this assessment of household contact networks, the contacts were matched based on the contact's name, age and gender. Household connectedness was calculated using two statistics assuming all non-reciprocal contacts were in fact reciprocal, i.e. for a given household size, the fraction of completely connected households and the mean network density, i.e. the average of the ratios of the number of observed contacts over the number of possible contacts was calculated. Household clustering was calculated as the proportion of contacts outside the household that made contact with two connected household members, averaged over all households of a given size. More specifically, household clustering was the clustering coefficient calculated from all triplets of two connected household members and a contact outside the household.[20] (See Supporting information for more details)

#### Exploration of the impact of household networks on disease transmission

We also explored the potential impact of the observed household networks on the transmission of diseases. For this, we assumed that mixing in the community happened at random, because of a lack of previous knowledge on intra and inter-community mixing in the study setting. We conducted epidemic simulations based on a two-level mixing epidemic model to quantify the impact of the assumption of random versus network-based mixing within study households while assuming background random mixing between households.[21] Various scenarios using different transmission rates within and between households were considered. For more details of this model we refer to the Supporting information.

## Results

We identified 9015 reported contacts from 588 study household members. The median age of respondents was 17 years (interquartile range [IQR] 4–34 years). Approximately 87% of respondents were at home during the first scheduled visit. The majority of interviews were conducted during weekdays (83%).

### Number of contacts and participant characteristics

**Fig 1:**
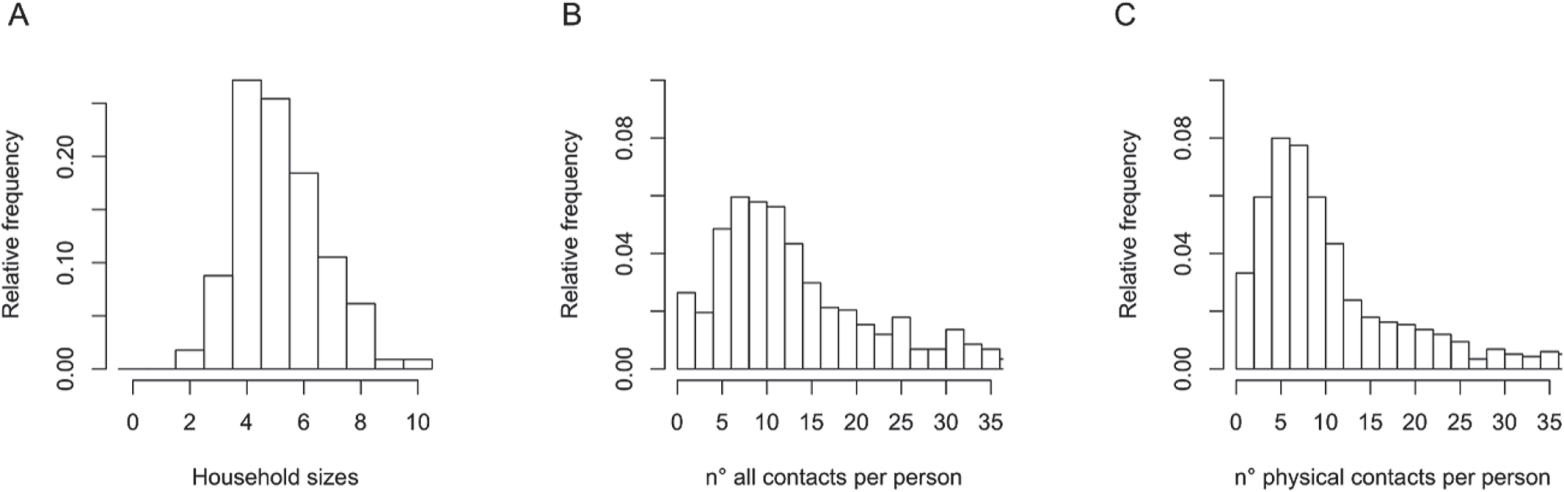
Distribution of household sizes (panel A). Distribution of the number of all (panel B) and physical (panel C) contacts per person per day ignoring household clustering.

The median number of reported contacts was 12 (IQR 8–20) whereas the median number of physical (skin-to-skin) contacts was 8.5 (IQR 5–14) (Fig. 1).

Table 1 shows the results of the conditional univariate, the conditional multivariate and the marginal model. Note that the multivariate conditional and marginal model, although warranting a different interpretation, yield very similar parameter estimates. Comparing results of the univariate and multivariate conditional models shows that taking as many participants' characteristics into account is important to obtain a correct interpretation of the relative effect (RE). Indeed, relationship to the index child and sex showed a significant effect on the number of contacts in the conditional univariate models but not in the multivariate models. This effect could be explained by collinearity between the different participant characteristics. For example, when dropping occupation from the model and adding sex to the model, sex became significantly associated with the number of contacts. This can be explained by the fact that most people working outside the home are men (92.2%) and most people working at home are women (73.9%). In summary, after accounting for the potential collinearity of model parameters, the number of reported contacts was mainly determined by the age of the participants, household size and occupation.

**Table 1:** Relative effects (RE) and 95% confidence intervals (CI) for the number of contacts based on univariate and multivariate conditional models and a multivariate marginal model.

Covariate (baseline: no)	Category	no	Univ. conditional model RE (95% CI)	Conditional model RE (95% CI)	Marginal model RE (95% CI)
Age	0–2	124	1.00	1.00	1.00
(years)	3–4	30	1.52 (1.23–1.88)	1.32 (0.97–1.81)	1.25 (1.01–1.56)
	5–9	61	2.38 (2.03–2.79)	1.48 (1.07–2.06)	1.41 (1.09–1.83)
	10–14	56	2.77 (2.36–3.27)	1.66 (1.19–2.31)	1.62 (1.26–2.08)
	15–19	36	2.04 (1.68–2.48)	1.50 (1.07–2.10)	1.55 (1.22–1.98)
	20–29	93	1.33 (1.16–1.54)	1.34 (0.95–1.90)	1.29 (1.04–1.61)
	30–59	139	1.30 (1.14–1.48)	1.31 (0.94–1.84)	1.28 (1.05–1.56)
	60–100	24	1.24 (0.98–1.58)	1.22 (0.83–1.79)	1.18 (0.86–1.62)
Sex	Male	270	1.00	1.00	1.00
	Female	293	0.86 (0.77–0.95)	0.91 (0.82–1.02)	0.93 (0.84–1.02)
Relationship	Index	113	1.00	1.00	1.00
(to Index)	Mother	112	1.26 (1.09–1.46)	0.97 (0.69–1.38)	0.98 (0.80–1.21)
	Father	87	1.34 (1.15–1.57)	0.84 (0.59–1.21)	0.88 (0.68–1.15)
	Sibling	162	2.22 (1.94–2.54)	0.89 (0.68–1.18)	0.88 (0.75–1.03)
	Grandparent	89	1.51 (1.28–1.78)	0.94 (0.68–1.29)	0.99 (0.78–1.27)
Occupation	At home	300	1.00	1.00	1.00
	Farmer	93	1.22 (1.09–1.38)	1.15 (0.94–1.41)	1.15 (0.96–1.38)
	School	128	2.38 (2.15–2.64)	1.91 (1.57–2.31)	1.97 (1.63–2.36)
	Other	42	1.27 (1.07–1.51)	1.19 (0.96–1.48)	1.17 (0.98–1.39)
Day	Weekday	469	1.00	1.00	1.00
	Weekend	94	0.84 (0.69–1.02)	1.00 (0.85–1.18)	0.97 (0.76–1.22)
Household size			1.11 (1.05–1.17)	1.05 (1.00–1.11)	1.05 (1.00–1.10)
Intercept				7.60	8.17
Overdispersion				6.51 (s.e. 0.64)	5.09 (s.e. 0.57)

Note that the overdispersion parameter is not shown for the univariate analyses and that REs for the conditional and marginal models are not comparable. All univariate conditional models showed significant overdispersion.

We repeated these analyses focusing on physical contacts and results were similar to those reported here, except for females making fewer physical contacts compared to males (univariate conditional model RE 0.73 (0.65–0.81), multivariate conditional model: RE 0.84 (0.75–0.94) and marginal model: RE 0.82 (0.74–0.92)).

### Contacts by location, duration and frequency

**Fig 2:**
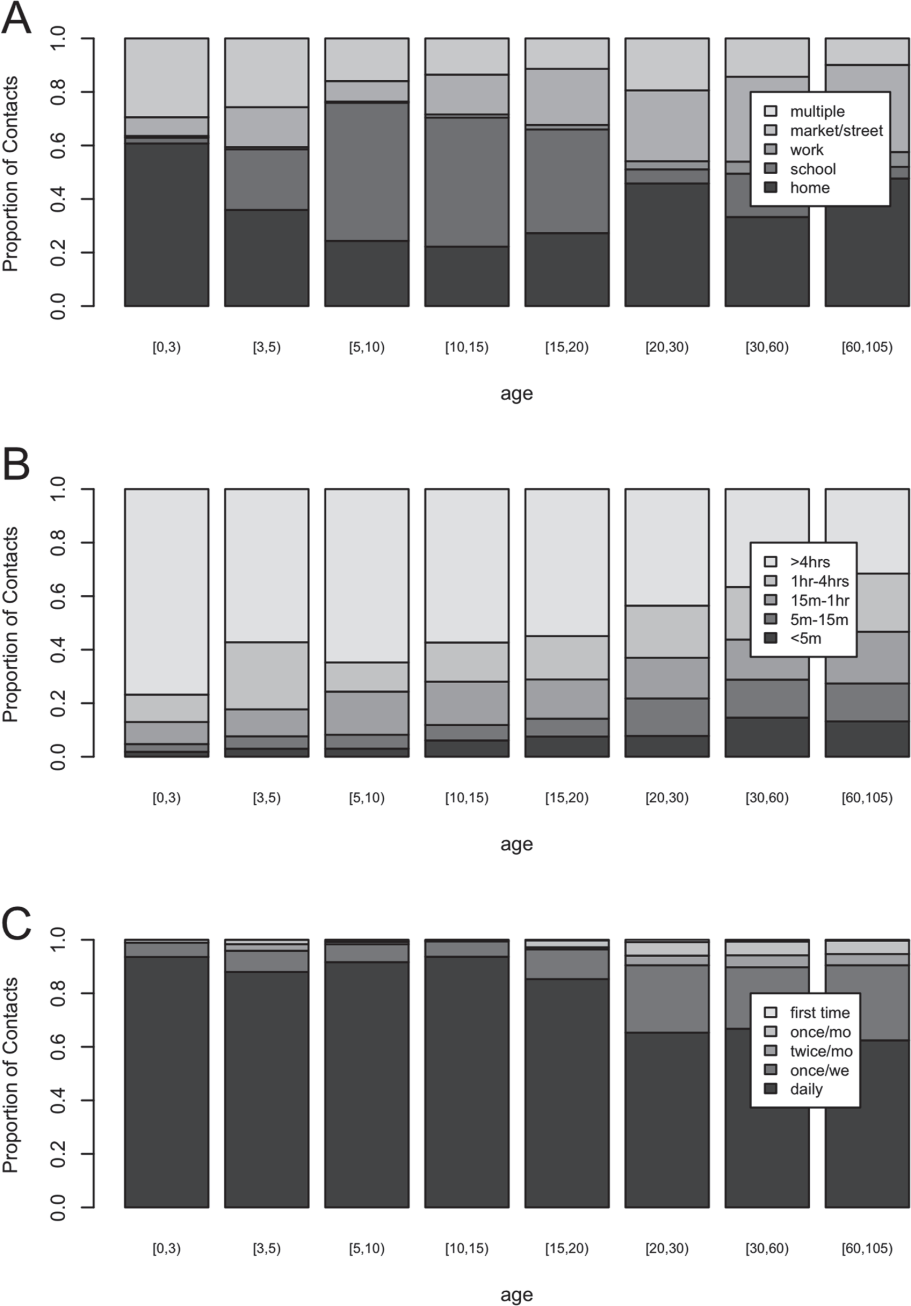
Proportion of contacts per category for location (panel A), duration (panel B) and frequency (panel C).

Fig. 2 shows the distribution of contact location (panel A), contact duration (panel B) and contact frequency (panel C) by age categories in years ([0,3), [3,5), [5,10], [10,15), [15,20), [20,30), [30,60), and 60+). For panel A, children 0 to 2 years old made most contacts at home, whereas participants 5 to 20 years old made most contacts at school. Participants reported few contacts at work whereas a substantial proportion of contacts was made at the market or street. Note that the category “multiple locations” refers to contacts made at more than one location, whereas all other reported contacts took place at one location only. Overall, participants reported a total of 1375 (15.3%) multiple location contacts with the most frequent combinations including contacts at home and at the market or street (62.0%), at home and at work (26.2%), at school and at the market or street (24.7%), and at home and at school (16.6%).

When focusing on contact duration, panel B in Fig. 2 shows that the proportion of long duration contacts decreases with participant's age, whereas the proportion of shorter duration contacts increases with participant’s age. Looking at contact frequency (panel C), daily contacts make up the majority of all contacts for all ages, with an increase in less frequent contacts for participants aged ≥15 years and especially ≥20 years. In summary, young children have more intense contacts and this intensity decreases with increasing age.

**Fig 3:**
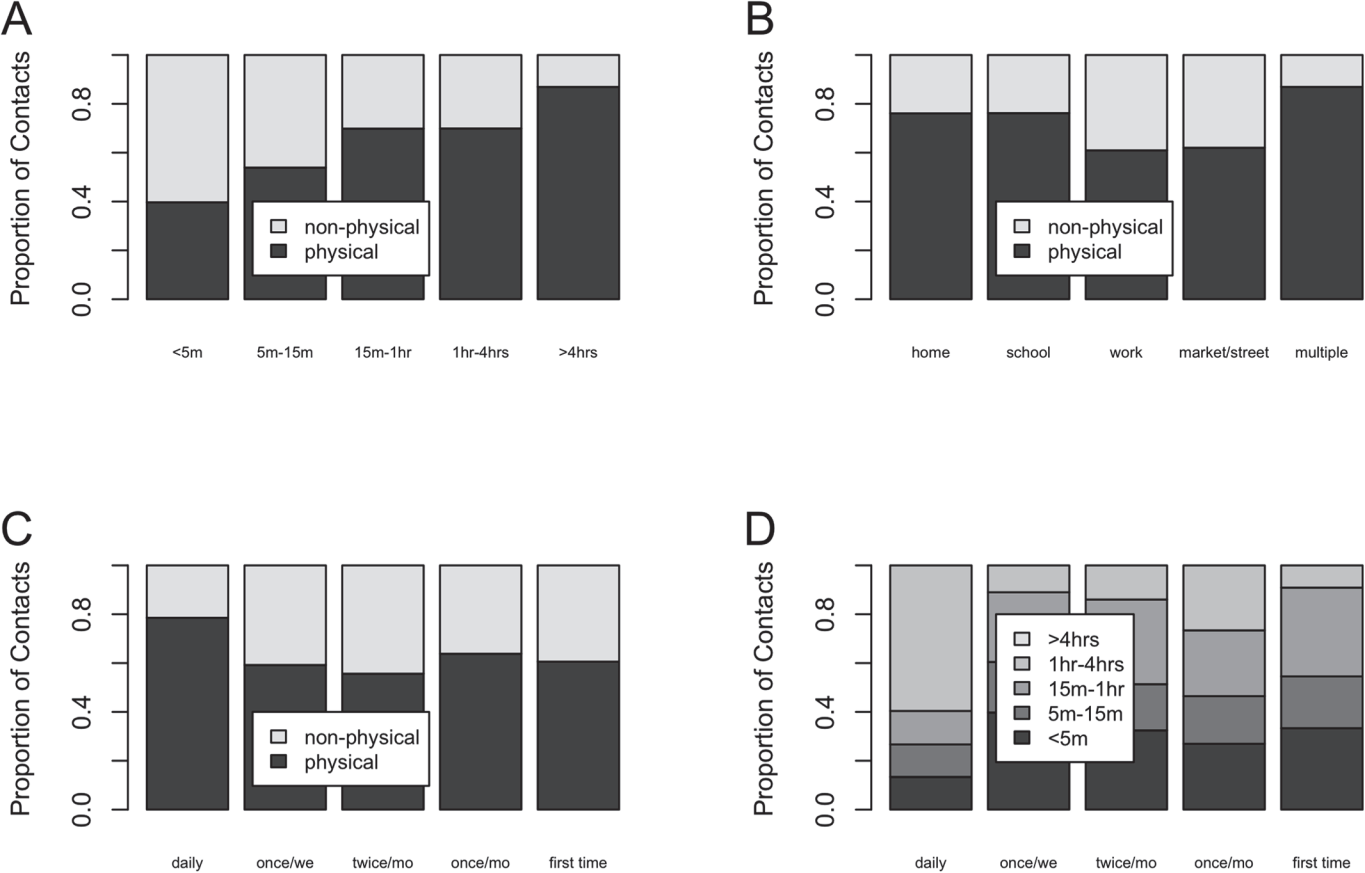
Proportion of physical and non-physical contacts by duration (panel A), location (panel B) and frequency (panel C). Proportion for the different categories of duration by frequency category (panel D).

Fig. 3 shows the proportion of non-physical and physical contacts by duration (panel A), location (panel B) and frequency (panel C) together with the proportion of different categories of contact duration by frequency (panel D). The proportion of physical contacts increases with contact duration, and the majority of physical contacts occurred at home and school. Similarly, the frequency of interactions with the contact person was positively associated with physical contact. Lastly, contacts occurring on a daily basis were more likely to last 4 hours or more.

### Who Acquires Infection From Whom?

**Fig 4:**
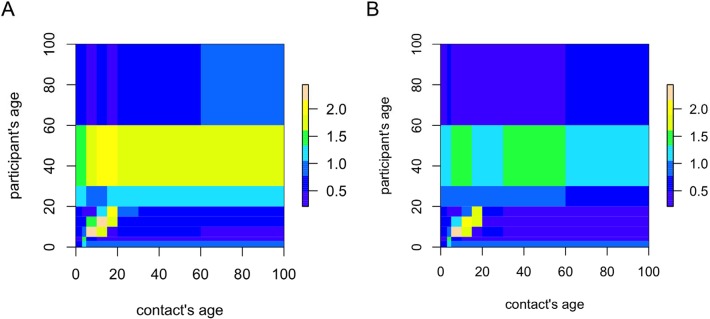
Logarithm of the mean number of contacts plus one for all recorded contacts (panel A) and for physical contacts only (panel B). Yellow indicates higher values while blue indicates lower values relative to the overall mean number of contacts.

Using age-categories [0,3), [3,5), [5,10], [10,15), [15,20), [20,30), [30,60), and 60+, we calculated the mean number of contacts m*_ij_* for all contacts and physical contacts, respectively. Fig. 4 illustrates the contact matrix log(m*_ij_* + 1) for all contacts (panel A) and physical contacts (panel B). These results indicate an assortative social mixing pattern for people aged <20 years whereas the mixing pattern is more uniform for people aged 20 years and above. The left panel of Fig. 4 shows that people aged 20–60 and 30–60 years also reported a relatively high mean number of contacts with children and teenagers, respectively, i.e. constituting intergenerational contacts. For the age group of 30–60 years, this is also pronounced when restricting to physical contacts only (Fig. 4, right panel). Note that the reverse, i.e. contacts with adults reported by children or teenagers, is not observed.

### Exploration of Household Contact Networks

Table 2 summarizes household network properties by household size. Household reciprocity seems to decrease with increasing household size. The fraction of completely connected households, assuming non-reciprocal links are in fact reciprocal, is generally high except for large households (≥7 members). The mean network density is high for all household sizes though its value is smaller for the household of size 9 for which one individual did not report any contacts with other household members. Clustering coefficients ranged from 0.328 to 0.861 with a majority of values larger than 0.5, but generally decreased with increasing household size. This suggests that contacts outside the household tended to be shared by household members and that clustering is important to consider when including household contact networks in transmission models.

**Table 2:** Household size frequency for all households and for households for which all members were sampled on the same day and reported contacts (eligible households), and important contact network characteristics based on these eligible households: reciprocity, connectedness (proportion complete, network density) and clustering.

Household size (number of members)	2	3	4	5	6	7	8	9
# eligible households/# households	2/2	6/10	20/31	11/29	8/21	4/12	2/7	1/1
Overall reciprocity	1.000	1.000	0.833	0.927	0.933	0.869	0.696	0.722
Proportion of completely connected households	1.000	1.000	0.950	0.909	1.000	0.750	0.000	0.000
Mean network density	1.000	1.000	0.992	0.998	1.000	0.988	0.946	0.778
Clustering	0.702	0.784	0.605*	0.449	0.531	0.409	0.328	0.861

*One household was excluded for this statistic given that no contacts outside the household were reported.

**Fig 5:**
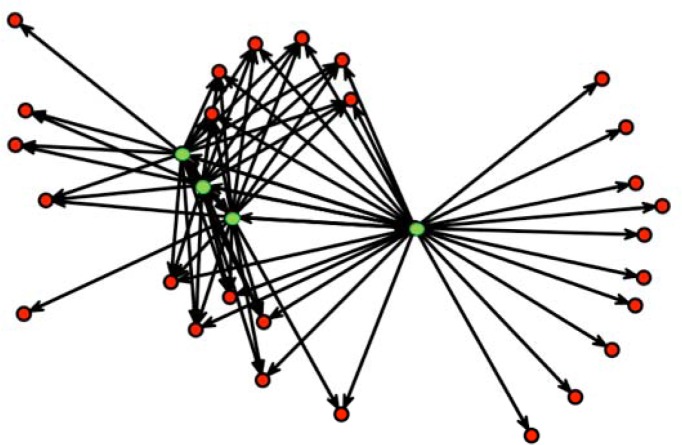
A directed contact network for a randomly selected household of size 4. Vertices are either household members (green) or contacts outside the household reported by these household members (red). Directed edges represent contacts as reported by household members.

For a randomly selected household of size 4, Fig. 5 illustrates the common interaction of household members with other non-household members, reflected by the relatively large clustering coefficient.

### Impact of household networks on disease transmission

**Fig 6:**
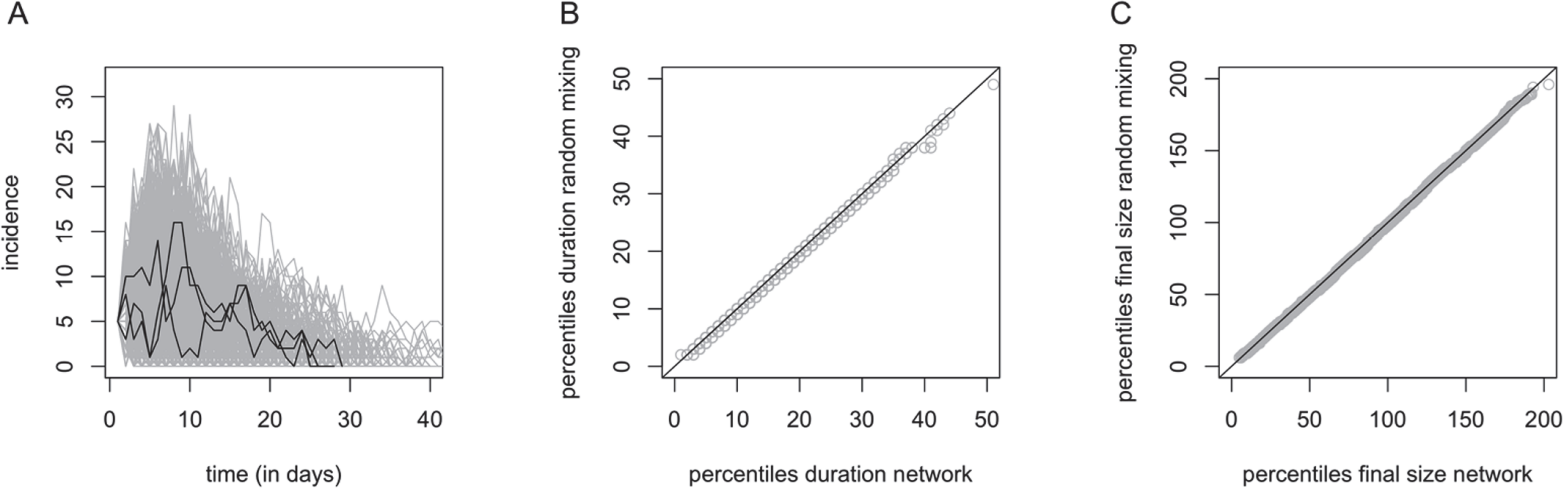
Findings from two-level mixing simulation models. Panel A: simulated epidemic curves (gray lines) using the observed household networks with three randomly chosen epidemic curves highlighted (black line). Panel B: scatterplot of the percentiles of the distribution of the duration of the simulated epidemics using the observed household networks (x-axis) and the completely connected households (y-axis). Panel C: scatterplot of the percentiles of the final size distribution of the simulated epidemics using the observed household networks (x-axis) and the completely connected households (y-axis).

Our comparison of simulated epidemics assuming within-household random mixing and within-household mixing based on the observed household networks did not reveal any distinguishable difference between the epidemic curves (Fig. 6, panel A: curves only displayed for the observed household networks), the size and duration of the simulated epidemics (Fig. 6, panels B and C). This comparison was based on 5000 simulated epidemics using a 2-level mixing model with 5 randomly distributed introductory cases. Other scenarios using a different number of introductory cases and/or different transmission rates within and between households yielded similar results (not shown).

## Discussion

We studied the underlying contact patterns upon which respiratory infections could spread in rural low-density community settings of the Peruvian Andes. In this household-based study, we observed a highly assortative pattern of social mixing where participants frequently interacted with other people of similar age, especially among children and adolescents. Interestingly, long duration and physical contacts were commonly reported by participants. Furthermore, the exploration of household networks suggest frequent and intense contacts among household members and common interaction with non-household members.

Compared with other contact assessments conducted in developed or semi-rural areas of developing countries,[1–3,12] the overall patterns of social mixing seem largely comparable. However, the proportion of frequent and physical contacts seems to be higher in the rural Andean communities. As infection transmission is likely favored by these types of intense interactions,[22] our observations are valuable to understand the burden and transmission patterns of respiratory infectious diseases in the study communities.

The optimal strategy for quantifying contact patterns remains unclear. Some investigators advocate prospective collection of contact data where participants record their encounters made during the study days. A few studies comparing prospective versus retrospective collection of information suggest that more contacts would be captured through a prospective data collection strategy when compared with a retrospective assessment.[3] Other evaluations did not find significant differences between these approaches.[23] Yet other studies have reported that retrospective collection of data would yield a larger number of contacts recorded.[24]

Although direct observation could provide an objective assessment of social mixing patterns in a given population, implementing such a measurement can be cumbersome and logistically challenging. Such a study design might be perceived as overly invasive and concerns about confidentiality might arise. This approach of direct observation may be better suited to closed environments where close monitoring could be accomplished provided the aforementioned concerns have been properly addressed. The recording of social interactions based on structured interviews and surveys is thus more feasible, at least currently. One advantage of our study is the use of a short and easy-to-administer study instrument that can be completed in an interview format. The same form has been applied previously in other urban locations and thus, the consistent use of a similar instrument facilitates the comparison of findings.[1,12] Furthermore, the form is detailed enough to enable the time-space characterization of the reported contacts.[3]

With regard to the household networks, our exploration identified an intense pattern of social mixing within study households, with a frequent potential for introduction of new infections into the households, although the actual spread of contagion was not evaluated. Interestingly, based on simulated epidemics, the observed pattern of within-household mixing in the study communities seems to be equivalent to the empirical random mixing assumed in previous simulation studies.[9,10] The conduct of similar household-based studies in other settings would be helpful to complement these observations.

Our assessment is subject to several limitations. Although we restricted our retrospective assessment to one day to minimize recall issues, recall could have still remained a problem. For example, some shorter encounters may have been forgotten.[25] Some participants may have reported contacts for an atypical day, for instance they may have been sick during the assessment, but we were not able to make that distinction in our study. Our data were collected within a few months, and roughly during the same season. Previous studies have suggested that social mixing may vary depending on the season and weather-related changes.[26] We focused on contacts including conversations and physical contacts but there may be other types of encounters relevant for transmission of infections that were not recorded (e.g. close proximity to other people during the weekend at open markets may be enough for transmission of pathogens without the need to establish a type of contact used in our study).[3] We also acknowledge that households participating in this study were selected based on convenience and on the presence of index children younger than 3 years of age and that the household network analyses were conducted in households for which all participants were at home on the day of the interview. Thus, these households may not be representative of the general population. So extrapolation of our findings to different settings requires careful consideration of our selection criteria. Nevertheless, our assessment provides novel information from an underserved population traditionally excluded from research. Ironically, this is a population that usually bears most of the brunt of mortality due to severe respiratory diseases.[14]

The study of the patterns of social interactions in rural Andean communities, suggest that contacts were highly assortative and appear to involve more physical contacts (especially at home and school) than previously described for other studies conducted in more developed and populated areas.[1–3,12] These observations can inform potential interventions including social distancing or other non-pharmaceutical interventions to mitigate the transmission of infectious diseases in these settings.

## Supporting Information

S1 Fig Age distribution of study participants and the underlying population of San Marcos. The dashed line represents the age distribution of the study population. The solid line represents the age distribution of the San Marcos population.
(TIFF)

S2 Fig Subgraph of order three with two household members (red nodes) and one non-household member (blue node). The solid blue lines make up a connected triplet whereas the dashed-dotted line shows the edge which determines whether the subgraph is a triangle (observed edge) or not (missing edge).
(TIF)

S1 Supporting Information Additional information about the methods used in the manuscript. (DOCX)

S1 Survey Form (PDF)
