# Supplementary figures and images for: A Household-Based Study of Contact Networks Relevant for the Spread of Infectious Diseases in the Highlands of Peru
Source: PLoS One. 2015 Mar 3;10(3):e0118457. doi: 10.1371/journal.pone.0118457 (PMC4348542; doi:10.1371/journal.pone.0118457)

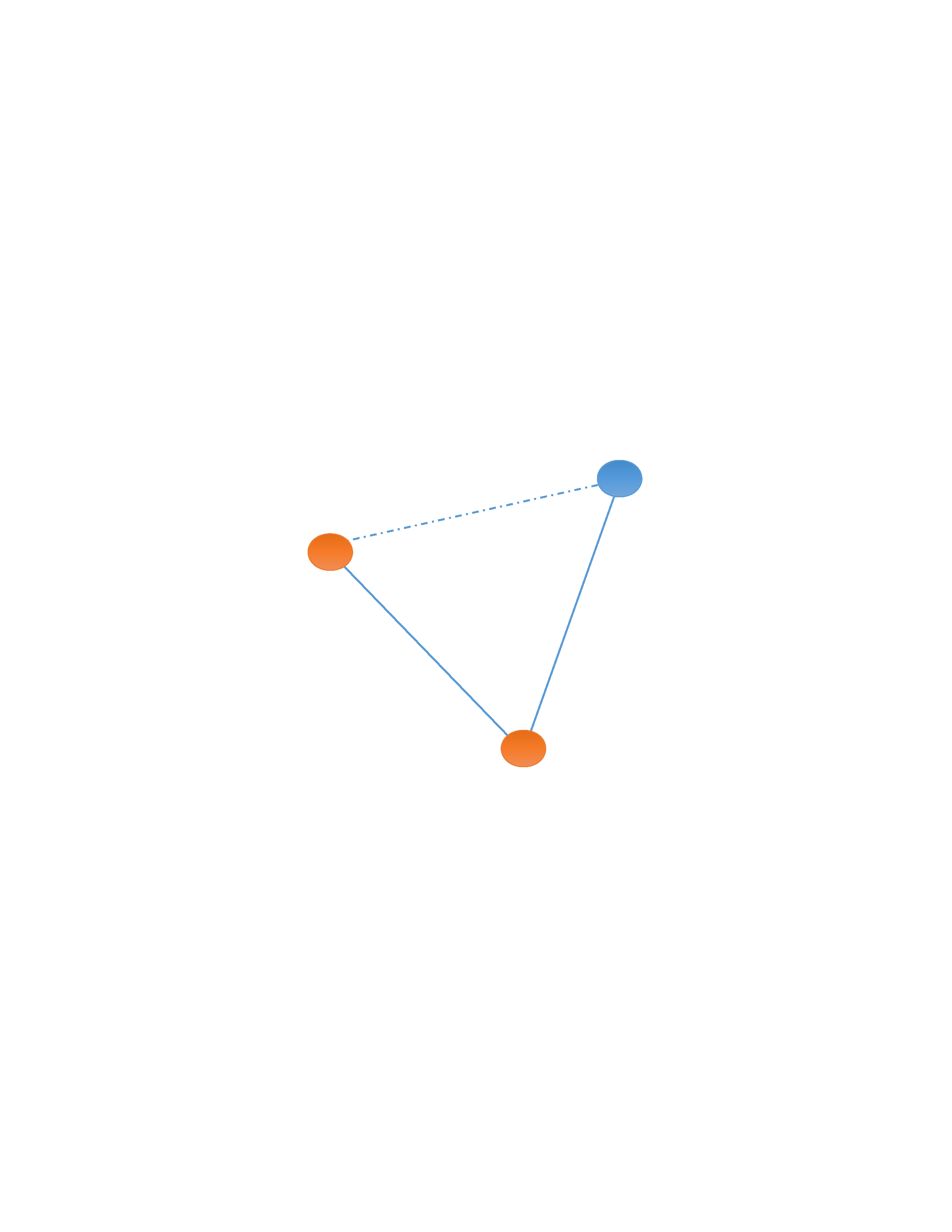

Supplement: S2 Fig — The solid blue lines make up a connected triplet whereas the dashed-dotted line shows the edge which determines whether the subgraph is a triangle (observed edge) or not (missing edge). (TIF) [file pone.0118457.s002.tif]
