# Supplementary material for: A Household-Based Study of Contact Networks Relevant for the Spread of Infectious Diseases in the Highlands of Peru
Source: PLoS One. 2015 Mar 3;10(3):e0118457. doi: 10.1371/journal.pone.0118457 (PMC4348542; doi:10.1371/journal.pone.0118457)
Supplement: S1 Supporting Information — (DOCX) [file pone.0118457.s003.docx]

**S1_Supporting information**

Additional information about the methods used in the manuscript

**Longitudinal regression models**

Accounting for the correlation among the number of contacts of individuals belonging to the same household requires the use of regression methods for correlated data. We used both a conditional and a marginal model warranting an individual-specific and a population-averaged interpretation, respectively. More specifically, the conditional model we used is a random intercepts model allowing for the estimation of household-specific levels of contact intensity. The marginal model we used is known as Generalized Estimating Equations (GEE) in which a sandwich estimator is used to correct variance estimation for the correlation between measurements from individuals within households.

**References**

Fitzmaurice, G.; Davidian, M.; Verbeke, G. & Molenberghs, G. (2009) Longitudinal Data Analysis CRC Press.

Molenberghs, G. & Verbeke, G. (2005) Models for Discrete Longitudinal Data Springer.

Zeger, S. & Liang, K. (1986) Longitudinal Data Analysis for Discrete and Continuous Outcomes Biometrics*, 42*, 121-130

Rue, H., Martino, S., and Chopin, N. (2009). Approximate Bayesian inference for latent Gaussian models using integrated nested Laplace approximations (with discussion). Journal of the Royal Statistical Society, Series B, 71(2):319–392.

**Clustering coefficient (network analysis)**

The clustering coefficient we use in our analysis was proposed by Bollobas and Riordan (2002) who improved its definition from the original version as proposed by Watts and Strogatz (1998) and can be expressed in terms of the density of triangles among connected triplets where a triangle is defined as a complete subgraph of order three, i.e. three nodes all connected to each other and a connected triple, i.e. three nodes connected by two edges (also called a 2-star). Note that within a triangle three connected triples can be identified. In our analysis we considered all subgraphs of order three as shown in Figure S2.

**References**

Watts, D. and Strogatz, S. (1998) Collective dynamics of ‘small-world’ networks. Nature, Vol 393, 6684, pp440-442.

Bollobas, B. and Riordan, O. (2002) Mathematical results on scale-free random graphs. In: Handbook of Graphs and Networks: From the Genome to the Internet, S. Bornholdt and H. Schuster, Eds. Weinheim: Wiley-VCH, pp 1-34

**The discrete-time chain binomial SIR model (Bailey 1957)**

We elaborate on the method proposed by Goeyvaerts et al (2014). We simulate the spread of a newly emerging infection in a closed fully susceptible population of households using a discrete-time chain binomial SIR model (Susceptible - Infected – Recovered, see Bailey (1957). We assume two levels of mixing similar to the households model of (Ball, 1997): high-intensity mixing within households and low-intensity `background' random mixing in the entire community. Two different configurations for within-household mixing are compared: the observed household configuration and a fully connected household configuration.

At time step $t$, each susceptible $i$ acquires infection with probability:

$$p_{i}\left( t \right)=1-\left( 1-\beta_{h} \right)^{\sum_{j\in h_{i},j\neq i} y_{ij}I_{j}\left( t \right)}\left( 1-\beta_{c} \right)^{\sum_{j\notin h_{i}} I_{j}\left( t \right)}$$

Where $\beta_{h}$ denotes the within-household transmission probability per contact, per time step and $\beta_{c}$ denotes the community-based transmission rate. Further, $h_{i}$ denotes the household of node$i$, $I_{j}(t)$ indicates whether node $j$ is infected (1) or not (0) at time $t$ and $y_{ij}=1$ if person $i$ and $j$ made contact and zero if not. .

We use several simplifying assumptions to study the impact of household network structure on the spread of infectious disease in this two-level mixing model. We assume that inherent susceptibility and infectiousness are invariant with age, that there is no latent period i.e. individuals are infectious immediately when acquiring infection and that infected nodes recover with a constant or infection-time-dependent probability.

Results are displayed for 5000 stochastic epidemic simulations with $\beta_{h}=0.01$ and $\beta_{c}=0.005.$

**References**

Goeyvaerts, N., Potter, G., Van Kerckhove, K., Willem, L., Beutels, P. and Hens, N. Exponential-family random graph models for within-household contact networks. International Biometric Conference, July 6-11, 2014, Florence, Italy. The IBC conference abstracts are publicly available at <http://www.ibs-italy.info/ibc-2014.html>. The complete presentation is available from <http://ibiostat.be/software>

**The R-code for the discrete-time chain binomial SIR model (Bailey 1957)**

# The call used to generate one epidemic using the function below. Note that we did not

# differentiate between children and adults though the function does allow for this.

epiChainBinModel(HH_network,beta1,beta2,gamma,nSeed)

# Epidemic model: discrete-time, chain binomial SIR model

# Author: Nele Goeyvaerts

# Version: 26/02/2014

# Standard chain binomial model with constant recovery probability

############################################################

## Input parameters:

# HH_network: static network object used for epidemic simulation

# (edge = event by which transmission may occur)

# beta1: within-household transmission probability per contact (link), per time step

# beta2: community transmission probability per contact (link), per time step

# gamma: parameter(s) specifying the duration of the infectious period

# recovery probability per time step

# nSeed: number of vertices that are seeded as infectious individuals

## Output parameters:

# timeSteps: duration of the epidemic (number of time steps)

# infectedMat: matrix indicating the current state (columns) for every vertex (rows)

# in the network: suceptible (0), infected (1) and recovered (NA)

# incidenceMat: matrix indicating whether a new infection occurred (rows) at

# each time step (columns)

# HH_network: static network object used for epidemic simulation

# (edge = event by which transmission may occur)

epiChainBinModel <- function(HH_network, beta1, beta2, gamma, nSeed){

# Parameters:

# Initial values

beta2 <- matrix(beta2, nrow = 2, ncol = 2)

nrVertices <- network.size(HH_network)

infected <- rep(0, nrVertices)

infected[sample(1:nrVertices, nSeed)] <- 1

timeSteps <- 1

incidence <- infected

infectedMat <- infected

incidenceMat <- incidence

# Individual attributes

hh_id <- HH_network %v% "hh_id"

vertexNames <- HH_network %v% "vertex.names"

age <- HH_network %v% "age"

child <- age <= 18

# Note: this is not equal to the "child" vertex attribute! Some children are older than 18

vertex_id <- 1:nrVertices

while(sum(infected, na.rm = TRUE) > 0){

# Repeat loop until all infected individuals are recovered and

# no new infections have occurred

# Infect each susceptible with a certain probability

incidence <- rep(0, nrVertices)

infectedPrev <- infected # indicator of infection in the previous time step

for(i in vertexNames[!is.na(infected) & infected == 0]){

select <- vertexNames == i

hh_contacts <- get.neighborhood(HH_network, vertex_id[select])

nrInfHhChild <- sum(infectedPrev[vertex_id %in% hh_contacts &

child == TRUE], na.rm = TRUE)

nrInfHhAdult <- sum(infectedPrev[vertex_id %in% hh_contacts &

child == FALSE], na.rm = TRUE)

nrInfHH <- nrInfHhChild + nrInfHhAdult

nrInfComChild <- sum(infectedPrev[child == TRUE], na.rm = TRUE)

- nrInfHhChild

nrInfComAdult <- sum(infectedPrev[child == FALSE], na.rm = TRUE)

- nrInfHhAdult

if(child[select] == TRUE){

infProb <- 1-(1-beta1)^nrInfHH*

(1-beta2[1,1])^nrInfComChild*(1-beta2[1,2])^nrInfComAdult

}else{

infProb <- 1-(1-beta1)^nrInfHH*

(1-beta2[2,2])^nrInfComAdult*(1-beta2[1,2])^nrInfComChild

}

infected[select] <- ifelse(runif(1) < infProb, 1, 0)

incidence[select] <- infected[select]

}

# Recover each infected with a certain probability

for(i in vertexNames[!is.na(infected) & infected == 1]){

infected[vertexNames == i] <- ifelse(runif(1) < gamma, NA, 1)

}

# Append current status to matrix and update time variable

infectedMat <- cbind(infectedMat, infected)

incidenceMat <- cbind(incidenceMat, incidence)

timeSteps <- timeSteps + 1

}

# List of output variables

return(list(timeSteps = timeSteps, infectedMat = infectedMat, incidenceMat =

incidenceMat, HH_network = HH_network))

}

**S1_Figure: Age distribution of study participants and the underlying population of San Marcos**

**
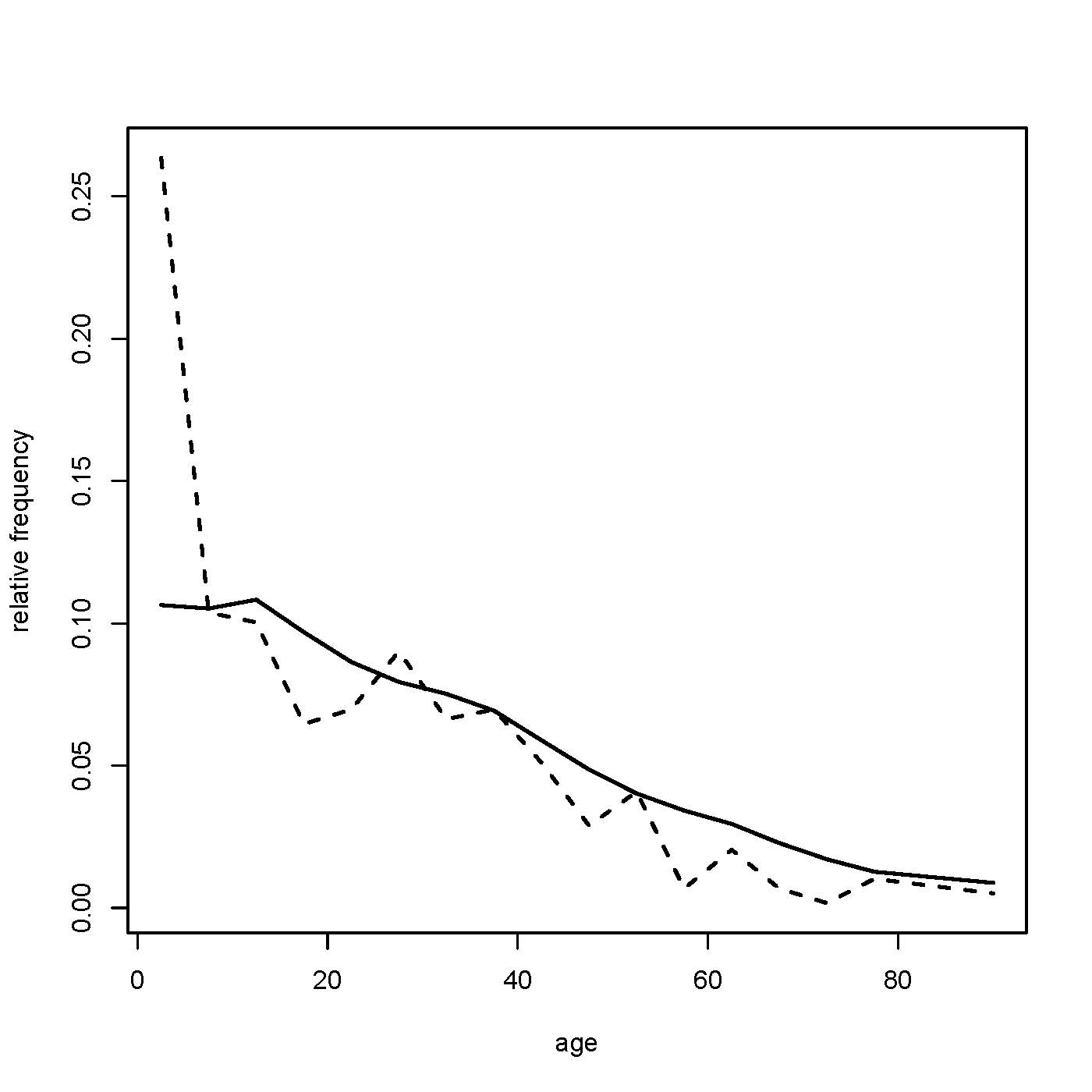
**

The dashed line represents the age distribution of the study population. The solid line represents the age distribution of the San Marcos population

**S2_Figure: Subgraph of order three with two household members (red nodes) and one non-household member (blue node)**

The solid blue lines make up a connected triple whereas the dashed-dotted line shows the edge which determines whether the subgraph is a triangle (observed edge) or not (missing edge).
