# Supplementary material for: A Household-Based Study of Contact Networks Relevant for the Spread of Infectious Diseases in the Highlands of Peru
Source: PLoS One. 2015 Mar 3;10(3):e0118457. doi: 10.1371/journal.pone.0118457 (PMC4348542; doi:10.1371/journal.pone.0118457)
Supplement: S1 Survey Form — (PDF) [file pone.0118457.s004.pdf]

Código Niño Enrolado : __ __ __ __

Proyecto: **Rol de la co-infección del virus de la influenza y del *Estreptococo pneumoniae* en infecciones respiratorias severas en niños**

FORMATO DEL ESTUDIO DE CONTACTOS

Comunidad No. __ __, Nombre: ______________________________

Nombre niño índice: ______________________________________

Encuestador: __ __

Fecha Encuesta: __ __ /__ __ / __ __ Hora inicio: __ __ : __ __ Hora fin: __ __ : __ __

Fecha de Segunda Encuesta: __ __/__ __ / __ __ Hora inicio: __ __ : __ __ Hora fin: __ __ : __ __ (llenar con 8 si no procede)

Texto para leerle al participante:

Sra., como Ud. recordará cuando le explicamos los procedimientos del proyecto, al momento que Ud. firmó el formato de consentimiento informado, le dijimos que en algún momento del proyecto le íbamos a preguntar a qué personas se ha visitado o se ha tenido contacto en el día anterior y si el niño ha dormido fuera de su hogar y con qué frecuencia. Quisiéramos ahora pedirle su participación en esta encuesta en donde vamos a preguntar a todas las personas que viven con Ud. en su hogar sobre ***todos*** *los contactos que haya tenido con otras personas durante el día antes de la encuesta. Esta encuesta va a ser muy útil para descubrir como los virus que hemos visto están causando enfermedades respiratorias en sus niños viajan de persona a persona. Le voy a pedir a cada persona que vive con Ud. en su hogar, que trate de recordar todos los contactos que va a tener entre las 5 de la mañana del día ________ hasta las 5 de la mañana del día ________. Yo voy a preguntar a todos los miembros de su hogar sobre todos los contactos ha tenido en el día indicado. Ud. me ayudara a recordar los contactos de los niños menores de 10 años. Para esta encuesta vamos pedirle a cada uno de Uds. recordar si ha existido un contacto con otras personas como es:*

- *Todas las personas que han estado cerca y que han conversado con cada uno de Ustedes (o sea han pronunciado 3 o más palabras con alguien de Uds.). En ese sentido no vamos a contar como un contacto a las personas con quien Ud. conversó por un teléfono celular, por ejemplo, pues no han estado cerca de Ud., así como a las personas con quien se habló pero que estaba a más de 3 metros de distancia; y*
- *Todas las personas que han tenido un contacto con Ustedes, o sea que su piel haya tocado la piel de la otra persona, como por ejemplo darle la mano, darle un abrazo, darle un beso, bañar a su hijo o cambiarle la ropa, etc.*

Queremos saber de ***todas las personas*** *con las que conversó o tuvo un contacto,* ***no importa si ha sido una persona que Ud. conoce o no (por ejemplo cuando viaja en un ómnibus o camioneta, o cuando está en una tienda o en el mercado) o por cuánto tiempo converso con la persona.***

Ahora voy a hacerle algunas preguntas iniciales. Desde ya le agradecemos por su apoyo en esta encuesta.

Encuesta Inicial

1. Cuántos miembros están viviendo con Ud. en su hogar? (comiendo de la misma olla y durmiendo en su hogar)

No.	Nombre	Edad años	Sexo (1=Masc, 2=Fem)	Parentesco (1=Madre, 2=Padre, 3= Hermano/a, 4=Abuelo/a materna, 5= Abuelo/a paterno, 6=Tio/a, 7=Otros (anotar))	Ocupación principal (1=su casa, 2=en la chacra, 3= vendiendo, 4=en la escuela, 5= otro (anotar)	Está presente en la casa? (0=No, 1=Si, 9=No sé)	Cuando puedo regresar para encuestar a esta persona? (anotar fecha y hora acordada)	Fecha de Encuesta Individual Realizada (dd/mm/aaaa)
1								
2								
3								
4								
5								
6								
7								
8								
9								
10								
11								
12								
13								
14								
15								
16								

2. Ud. o alguien de su familia vende algún producto o animal en el mercado o feria semanal? 2. __
   (0=No, 1=Si)
3. Acude Ud. o alguien de su familia al mercado o feria semanal? 3.__
   (0=No, 1=Si) Si la respuesta es no, pasar a la pregunta 6 llenando con 8 (no procede) las preguntas 4 y 5
4. A cuál? Anotar el nombre: ________________________________
5. Lleva con Ud. al niño al mercado o feria? 5.__
   (0=No, 1=Si, 8= No procede).
6. Durante el último mes, ha dormido el niño fuera de su hogar al menos una noche? 6.__
   (0=No, 1=Si, 9=No recuerdo, no se) En caso de No, o No se, terminar la encuesta inicial.

7. Me podría decir donde durmió el niño fuera de su hogar en el último mes?

Lugar<br>Anotar el nombre de ciudad, o lugar en el campo	En casa de quién?<br>1=familiar, 2=hotel, 3=amigos, 4=casa propia 5= otro	Cuantas noches	Más o menos cuantas personas durmieron con Uds en esa casa?	Había alguien enfermo con tos, mocos, fiebre en esa casa?<br>0=No, 1=Si

Código Niño Enrolado : __ __ __ __ **Nro Encuestado : __ __**

Nº de miembro: ________ Nombre del miembro del hogar:________________________

Contacto No.	Nombre	Edad (Años. Ver nota)	Sexo 1=Fem 2=Masc	Le toco la piel? 1=Si 0=No	Con que frecuencia tiene contacto con esta persona? (ver códigos)	Donde tuvo contacto con la persona? Anotar todo lo que aplique durante el día							Tiempo en total que paso en contacto con la persona (conversando o contacto con piel) Ver códigos
						En su casa 1=Si, 0=No	Escuela Colegio 1=Si, 0=No	Trabajo 1=Si, 0=No	Feria / Mercado 1=Si, 0=No	Calle (diferente a la feria) 1=Si, 0=No	Transporte 1=Si, 0=No	Otro (indicar) 1=Si, 0=No	
01													
02													
03													
04													
05													
06													
07													
08													
09													
10													
11													
12													
13													
14													
15													
16													
17													
18													
19													
20													

Notas:

Edad en años: colocar los años del contacto si se conoce, o el rango de edad de grupos de 5 años: ejemplo de 10 a 14, de 15 a 19, de 20 a 24, de 25 a 29, etc.
Frecuencia del contacto: 1= Diario o casi a diario, 2= Una o dos veces por semana, 3= Una o dos veces por mes, 4= Menos de una vez por mes, 5= Nunca lo había conocido
Tiempo de contacto: 1=Menos de 5 minutos, 2= de 5 a 15 minutos, 3= de 15 minutos a una hora, 4= de 1 a 4 horas, 5= más de 4 horas.

 encuesta individual

Código Niño Enrolado : __ __ __ __ **Nro Encuestado : __ __**

Contacto No.	Nombre	Edad (Años. Ver nota)	Sexo 1=Fem 2=Masc	Le toco la piel? 1=Si 0=No	Con que frecuencia tiene contacto con esta persona? (ver códigos)	Donde tuvo contacto con la persona? Anotar todo lo que aplique durante el día							Tiempo en total que paso en contacto con la persona (conversando o contacto con piel) Ver códigos
						En su casa 1=Si, 0=No	Escuela Colegio 1=Si, 0=No	Trabajo 1=Si, 0=No	Feria / Mercado 1=Si, 0=No	Calle (diferente a la feria) 1=Si, 0=No	Transporte 1=Si, 0=No	Otro (indicar) 1=Si, 0=No	
21													
22													
23													
24													
25													
26													
27													
28													
29													
30													
31													
32													
33													
34													
35													
36													
37													
38													
39													
40													

Notas:

Edad en años: colocar los años del contacto si se conoce, o el rango de edad de grupos de 5 años: ejemplo de 10 a 14, de 15 a 19, de 20 a 24, de 25 a 29, etc.
Frecuencia del contacto: 1= Diario o casi a diario, 2= Una o dos veces por semana, 3= Una o dos veces por mes, 4= Menos de una vez por mes, 5= Nunca lo había conocido
Tiempo de contacto: 1=Menos de 5 minutos, 2= de 5 a 15 minutos, 3= de 15 minutos a una hora, 4= de 1 a 4 horas, 5= más de 4 horas.

Código Niño Enrolado : __ __ __ __ **Nro Encuestado : __ __**

Contacto No.	Nombre	Edad (Años. Ver nota)	Sexo 1=Fem 2=Masc	Le toco la piel? 1=Si 0=No	Con que frecuencia tiene contacto con esta persona? (ver códigos)	Donde tuvo contacto con la persona? Anotar todo lo que aplique durante el día							Tiempo en total que paso en contacto con la persona (conversando o contacto con piel) Ver códigos
						En su casa 1=Si, 0=No	Escuela Colegio 1=Si, 0=No	Trabajo 1=Si, 0=No	Feria / Mercado 1=Si, 0=No	Calle (diferente a la feria) 1=Si, 0=No	Transporte 1=Si, 0=No	Otro (indicar) 1=Si, 0=No	
41													
42													
43													
44													
45													
46													
47													
48													
49													
50													
51													
52													
53													
54													
55													
56													
57													
58													
59													
60													

Notas:

Edad en años: colocar los años del contacto si se conoce, o el rango de edad de grupos de 5 años: ejemplo de 10 a 14, de 15 a 19, de 20 a 24, de 25 a 29, etc.
Frecuencia del contacto: 1= Diario o casi a diario, 2= Una o dos veces por semana, 3= Una o dos veces por mes, 4= Menos de una vez por mes, 5= Nunca lo había conocido
Tiempo de contacto: 1=Menos de 5 minutos, 2= de 5 a 15 minutos, 3= de 15 minutos a una hora, 4= de 1 a 4 horas, 5= más de 4 horas.

 encuesta individual

Considera Ud. que ayer fue un día normal o no? (seleccione una opción):

- Fue un día normal, no estaba enfermo
- Me sentí enfermo(a) (cualquier enfermedad)
- No fue un día normal, no estaba enfermo pero hice cosas que normalmente no hago todos los días

Por favor asegúrese que no haya contactos no registrados
Si por alguna razón no puede incluir todos los contactos (por ejemplo si tiene una tienda y tiene que atender a mucha gente), por favor indicarlo:

☐ **He incluido todos los contactos que he tenido**

☐ **No he incluido todos los contactos que he tenido**

Si no ha incluido todos los contactos que ha tenido, aproximadamente cuanta gente no fue registrada y más o menos q edad tenían?

**Conversaciones con otras personas (sin contacto piel con piel):**	
**Grupo Etario**	Persona :
0-4 años	
5-9 años	
10-14 años	
15-19 años	
20-24 años	
25-29 años	
30-34 años	
35-40 años	
40-49 años	
50-59 años	
60 o más años	
**Contacto piel con piel:**	
0-4 años	
5-9 años	
10-14 años	
15-19 años	
20-24 años	
25-29 años	
30-34 años	
35-40 años	
40-49 años	
50-59 años	
60 o más años	

Casos especiales: niños en escuela o guarderías (wawawasi)

Los niños que van a la escuela o colegios o se encuentran en guarderías y que no son observados durante todo el día por la madre/padre y por lo tanto, **sus contactos durante el tiempo que están en la escuela** no pueden ser evaluados con la estrategia descrita anteriormente, se debe identificar a las personas con quien el niño estuvo en contacto más estrecho (compañero de carpeta, amigos con quien juego, amigo con quien fue a la escuela, etc) y agregar a esas personas en la lista de contactos del niño/a. Para contactos más generales y difusos, como personas en el recreo con quien se compartió un espacio, como jugando, etc, se llena la tabla de contactos generales, estimando el número de personas con contacto y contacto de piel a piel.

Al término de la encuesta agradecer a todos los miembros por su participación. Asegurar a todos que la información colectada será tratada en forma estrictamente confidencial

 encuesta individual
